# Sirolimus in a Renal Transplant Recipient Infected With COVID-19: A Blessing in Disguise?

**DOI:** 10.7759/cureus.17102

**Published:** 2021-08-11

**Authors:** Dhruv Talwar, Sunil Kumar, Sourya Acharya, Vidyashree Hulkoti, Akhilesh Annadatha

**Affiliations:** 1 Department of Medicine, Jawaharlal Nehru Medical College, Datta Meghe Institute of Medical Sciences (Deemed to be University), Wardha, IND

**Keywords:** mtor inhibitors, covid19, renal transplant, chronic kidney disease, immunocompromised

## Abstract

Immunocompromised status Is often associated with severe coronavirus infection given the inability of the immune system to combat the deadly severe acute respiratory syndrome coronavirus 2 (SARS-CoV-2). Patients with multiple comorbidities such as diabetes mellitus, hypertension, and chronic kidney disease along with patients on immunosuppressants or chemotherapy are at higher risk of getting infected during the ongoing pandemic with more probability of adverse outcomes. However, we report a rare case of a renal transplant recipient who was on sirolimus and contracted coronavirus disease (COVID-19). His immunosuppressants were continued and he was managed with antiviral, steroids and low molecular weight heparin and the patient responded well to the treatment and recovered completely after a span of one week. Use of sirolimus in a patient with renal transplant recipient helped in preventing intensification of the severity in COVID-19 attributing to its inhibiting effect on mammalian target of rapamycin (mTOR) which he was using post his renal transplant, therefore, proving to be a blessing in disguise.

## Introduction

Coronavirus infectious disease has introduced a lethal catastrophe around the world [1]. While the entire population is at risk of contracting this life-threatening disease, Immunocompromised individuals such as solid organ transplant (SOT) recipients are at greater risk of contracting the disease [2]. Though studies regarding the effect of coronavirus disease (COVID-19) in solid organ transplant recipients are still underway, premature data suggest a greater severity of COVID-19 in such patients. Immunosuppression provided in renal transplant recipients remains the primary cause for such adverse outcomes with COVID-19; however, the use of mammalian target of rapamycin (mTOR) inhibiting drugs might change the course of COVID-19 disease in solid organ recipients. There have been studies investigating the use of mTOR inhibitors as antiviral agents which might prove to be beneficial in influenza and other viral illnesses, including COVID-19. mTOR inhibiting drugs are used to inhibit the mechanistic target of rapamycin which controls the metabolism, proliferation, and growth of the cells. Sirolimus is an mTOR inhibiting cytostatic drug that is used in solid organ transplant recipients to prevent rejection of the transplanted organ. It is also used in stents used in coronary artery disease. Ever since the beginning of the COVID-19 spread, there have been numerous attempts to understand the complex mechanism of COVID-19 disease and to develop a definitive treatment for the same [3]. However, no single modality of treatment has been established as an assured treatment of choice. Anti-inflammatory effects of steroids have shown promising results in treating the cytokine storm associated with rapid deterioration of COVID-19 patients [4]. Certain immunomodulators are also showing promising results in the treatment of COVID-19 but only with carefully selected patients. It is important to study the unestablished relationship between mTOR inhibitors and COVID-19 as their continuation or discontinuation during COVID infection can be a tricky decision for the treating physicians.

## Case presentation

**Figure 1:**
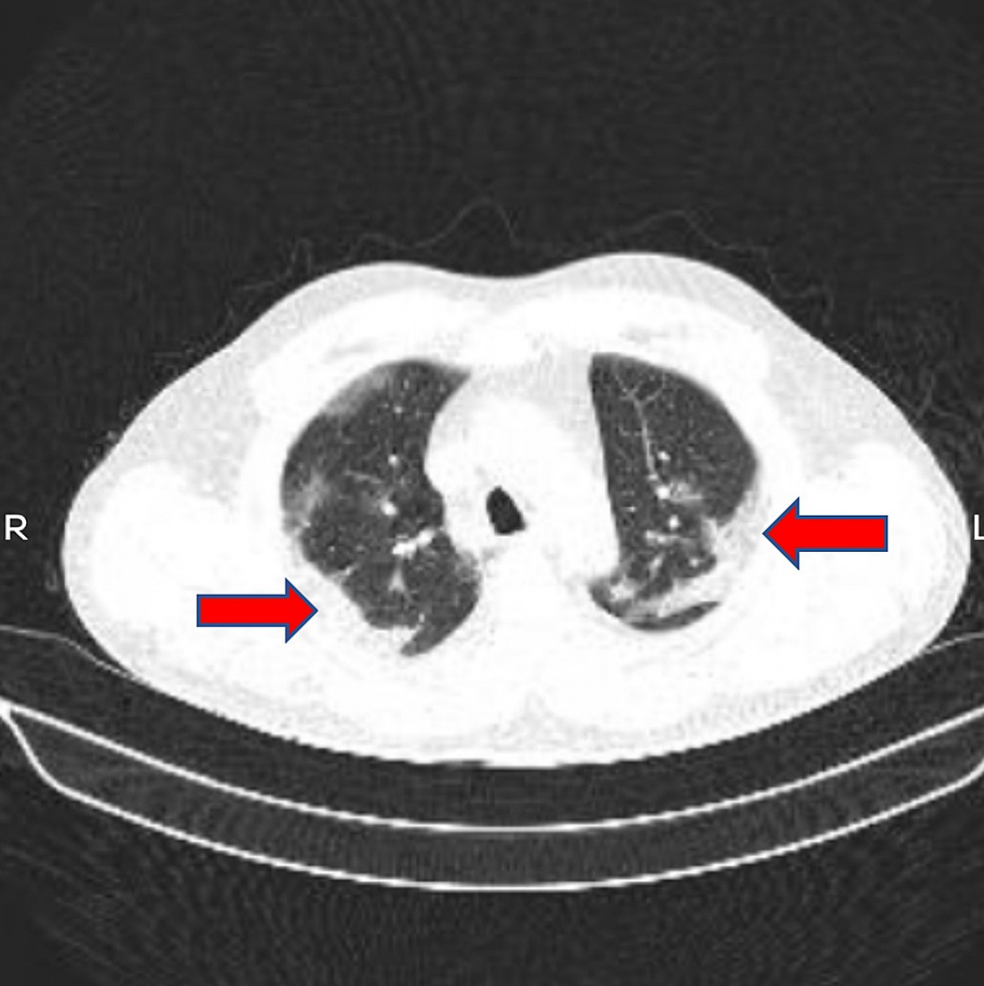
Showing HRCT chest with bilateral ground glass opacities predominantly in the lower lobes signifying COVID pneumonia. HRCT: High-resolution computerized tomography.

A 38-year-old male presented with the chief complaint of fever for three days along with dry cough. He had a history of contact with a COVID-19 patient seven days before he developed symptoms. There was a history of renal transplant three years back in view of diabetic nephropathy for which he was on sirolimus along with steroids. There was a history of diabetes mellitus for which the patient was regularly taking Insulin. There was no history of any other chronic illness and patient had no history of smoking. On general examination his pulse was 112/min regular in rhythm and volume, blood pressure was 140/88 mmHg on the right arm in the supine posture, Spo2 was 93% on room air and respiratory rate was 26 breaths per minute. Clinical examination was suggestive of pneumonia. The patient was admitted and a nasopharyngeal swab for COVID-19 was conducted via reverse transcriptase-polymerase chain reaction method which came out to be positive. High-resolution computerized tomography (HRCT) chest was done which showed bilateral ground-glass opacities suggestive of viral pneumonia with a CT severity score of 7/25 and CORAD 6 (Figure 1). Lab investigations showed normal complete blood count, liver function test with a raised creatinine, urea, D-dimer, CRP, ferritin, lactate dehydrogenase and an HbA1c of 7.1 (Table 1). The patient was started on remdesivir and was continued on sirolimus and steroids. The patient was monitored daily for spo2 levels and fever spikes or any aggravation of symptoms. His clinical condition improved markedly and he was discharged in a stable condition after seven days of hospitalization. He is currently doing well on follow-up.

**Table 1:** Showing lab investigations of the case.

Lab parameter	Result
Interleukin 6	67 pg/ml
Serum ferritin	601 g/ml
Lactate dehydrogenase	710 u/ml
C reactive protein	62 mg/l
Hemoglobin	10.3 gm/dl
Mean corpuscular volume	81 fl
Platelet count	97000/dl
White blood cell count	6800/dl
Creatinine	2.9 mg/dl
Urea	50 mg/dl
Sodium	138 mmol/l
Potassium	4.9 mmol/l
Albumin	2.9 gm/dl
Hemoglobin A1C (HbA1c)	7.1

## Discussion

**Figure 2:**
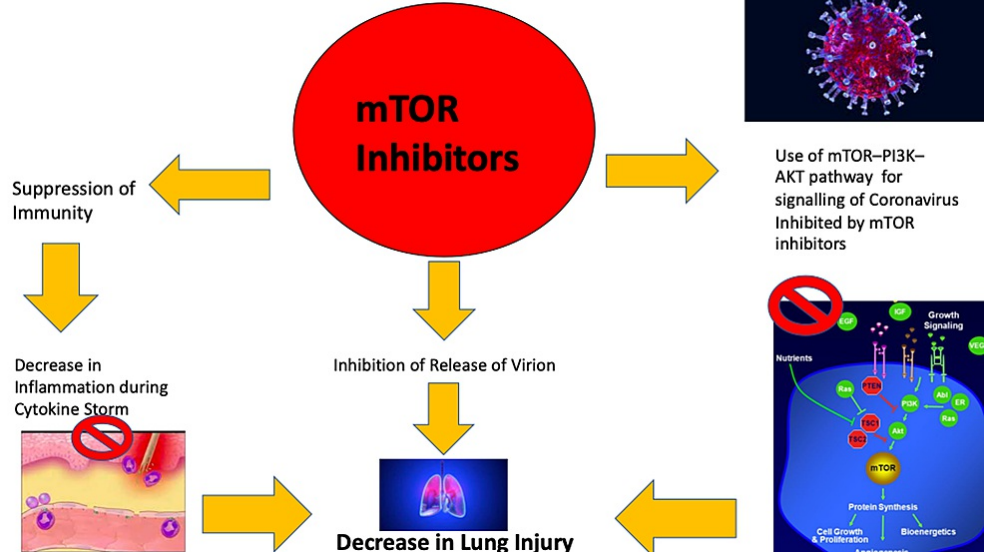
Mechanism of mitigating lung injury by mTOR inhibitors. mTOR: mammalian target of rapamycin.

COVID-19 in organ transplant recipients remains a domain of active research. The first case of a solid organ transplant recipient was reported from China where two patients who had previously had undergone heart transplantation contracted COVID-19 [5]. A cohort study conducted in Italy reported a mortality rate of twenty-five percent in a renal transplant patient with COVID19 concluding unfavorable outcomes in COVID-19 patients who had a history of renal transplant [6]. Also as mTOR inhibiting drugs are not always used as first-line drugs for renal transplant recipients, patients receiving mTOR inhibitors are underrepresented in studies based on solid organ transplant recipients with COVID-19 infection. Long-term consequences of COVID-19 in renal transplant recipients are yet to be seen. With the limited research on solid organ transplant patients with COVID-19, it is difficult to establish a definitive relationship between the history of Organ Transplantation and the outcome of COVID-19 but it is reasonable to presume that due to the dysregulation of immunity Solid organ transplant patients are associated with the unfavourable outcome with COVID-19 [7]. Immunosuppression in COVID-19 is a controversial topic owing to the two phases during the course of the disease. This first phase comprises viremia and the second phase deals with inflammation. The second phase of the disease deals with the activation of T cells leading to a storm of cytokines in the circulation ultimately resulting in acute injury of the lung, while immunosuppressives may cause increased viremia it might prove to help suppress the widespread systemic inflammation in the second phase of the disease thereby preventing acute lung injury. Discontinuing medication like sirolimus during the inflammatory phase would have resulted in systemic inflammation, extensive lung injury, and hypoxia [8]. Thus, in our case, as the inflammatory markers were increased we decided to continue the patient on sirolimus and steroids. Sirolimus was also found to stop the expression of proteins present in the virus along with inhibiting the release of virion concerning MERS-CoV. There is a similarity in the structure of severe acute respiratory syndrome coronavirus 2 and MERS-CoV and SARS-CoV-2 is using the mTOR-PI3K-AKT pathway as a primary signaling pathway. Therefore, the antiviral and immunosuppressive properties of mTOR inhibitor showed a benefit of benign disease course in a patient with multiple predictors of severe COVID such as co-morbidities like Diabetes along with the history of solid organ transplantation. Our postulate is supported by previous research showing antiviral properties in mTOR inhibitors not only in MERS CoV but also SARS-CoV-2 along with the severe inflammation witnessed in COVID-19 which might be tackled by immunosuppressive effects of mTOR inhibitors (Figure 2). Therefore, mTOR inhibitors have a theoretically sound mechanism of action in reducing the severity of COVID-19 infection which is yet to be proven and requires further studies given the contradictory findings in different studies conducted to date.

## Conclusions

We conclude that mTOR inhibitors in SOT recipients can show promising results in combat against COVID-19 provided they are continued after proper assessment and risk-benefit ratio analysis. While immunosuppressive agents promote viremia in the initial phase they might prove to be a blessing in disguise during the second phase of systemic inflammation of COVID owing to their anti-inflammatory effects.
